# Supplementary material for: Heat-Treated Micronized Polyethylene Powder for Efficient Oil/Water Separating Filters
Source: Materials (Basel). 2020 Jul 15;13(14):3160. doi: 10.3390/ma13143160 (PMC7412253; doi:10.3390/ma13143160)
Supplement: Supplementary file 1 [file materials-13-03160-s001.pdf]

materials

Supplementary Materials

Heat-Treated Micronized Polyethylene Powder for Efficient Oil/Water Separating Filters

Yasmin A. Mehanna [1,2] and Colin R. Crick [2,*]

[1] Materials Innovation Factory, Department of Chemistry, University of Liverpool, Liverpool L69 7ZD, UK; Yasmin.Mehanna@liverpool.ac.uk

[2] School of Engineering and Materials Science, Queen Mary University of London, Mile End Road, London E1 4NS, UK

* Correspondence: c.crick@qmul.ac.uk

Received: 12 June 2020; Accepted: 13 July 2020; Published: 15 July 2020

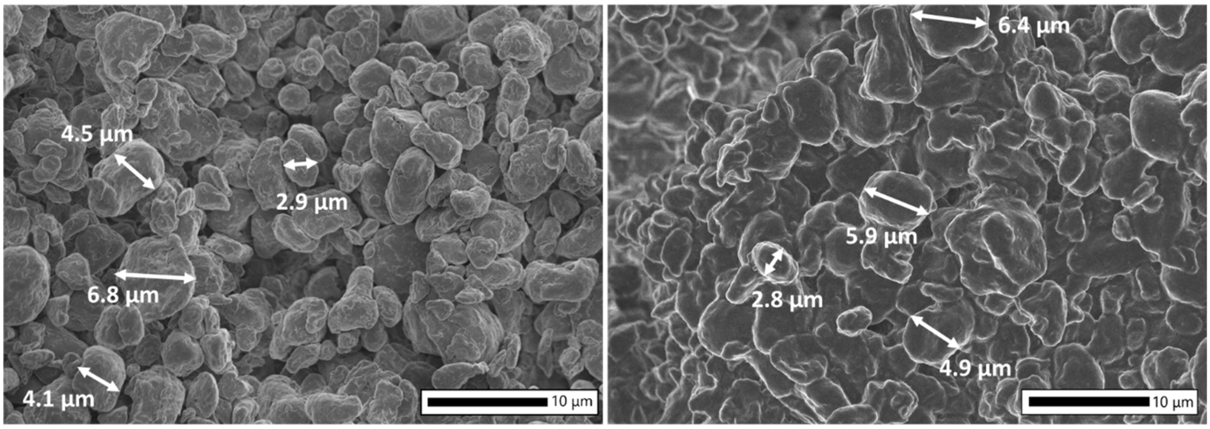

Figure S1. SEM images show S-μ PE powder with no solvent/heat treatment (**left**), and powder dispersed in hexane, sprayed and heated at 114°C (**right**). While both solvent and heat-treatment facilitate particles adhesion, the overall particle sizes do not appear to change during this process. This leads us to conclude that the dispersion/spraying process has probably no or very little impact on the average particle size.

Table S1. Tilt angles measured on PE filters, made with powder sizes: L-μ, M-μ and S-μ.

Trial	*L-μ*	*M-μ*	*S-μ*
1	21.39	14.52	8.71
2	25.42	11.87	8.68
3	25.2	12.85	9.74
4	20.41	13.38	11.71
5	17.45	14.24	10.21
6	19.89	12.97	10.99
7	22.11	14.01	12.5
8	21.76	13.31	12.54
9	22.18	11.95	10.62
10	19.19	12.19	11.61
Average	21.50	13.129	10.731
Error	2.48	0.94	1.40

Table S2. Tilt angles measured on PE secondary layer sprayed on glass slide, made with powder sizes: M-μ and S-μ.

Trial	***M-μ***	***S-μ***
1	3.69	5.07
2	6.29	4.82
3	8.53	9.21
4	7.58	8.93
5	3.27	7.49
6	5.08	12.03
7	4.42	6.16
8	3.84	11.37
9	5.63	11.48
10	7.25	3.63
11	5.09	8.6
12	6.35	5.28
13	4.37	11.5
14	4.04	5.87
15	9.09	4.66
16	4.12	9.55
17	7.07	9.01
18	4.33	7.77
19	8.67	10.27
20	4.13	6.23
21	7.95	5.83
22	5.64	8.87
23	5.68	5.92
24	5.07	7.42
Average	5.72	7.79
Error	1.74	2.47

© 2019 by the authors. Submitted for possible open access publication under the terms and conditions of the Creative Commons Attribution (CC BY) license (http://creativecommons.org/licenses/by/4.0/).
